# Long-term imaging and spatio-temporal control of living cells using targeted light based on closed-loop feedback

**DOI:** 10.1007/s12213-024-00165-0

**Published:** 2024-04-12

**Authors:** Neshika Wijewardhane, Ana Rubio Denniss, Matthew Uppington, Helmut Hauser, Thomas E. Gorochowski, Eugenia Piddini, Sabine Hauert

**Affiliations:** 1 Centre for Doctoral Training in Digital Health and Care, University of Bristol, Bristol, UK; 2 School of Engineering Mathematics and Technology, University of Bristol, Bristol, UK; 3 School of Cellular and Molecular Medicine, University of Bristol, Bristol, UK; 4 Bristol Robotics Laboratory, University of Bristol, University of West of England, Bristol, UK; 5 Centre for Doctoral Training in FARSCOPE, University of Bristol, University of West of England, Bristol, UK; 6 School of Biological Science, University of Bristol, Bristol, UK; 7 Bristol Synthetic Biology Research Centre, University of Bristol, Bristol, UK

**Keywords:** Targeted light, Closed-loop control, Live cell imaging

## Abstract

The ability to optically interact with cells on both an individual and collective level has applications from wound healing to cancer treatment. Building systems that can facilitate both localised light illumination and visualisation of cells can, however, be challenging and costly. This work takes the Dynamic Optical MicroEnvironment (DOME), an existing platform for the closed-loop optical control of microscale agents, and adapts the design to support live-cell imaging. Through modifications made to the imaging and projection systems within the DOME, a significantly higher resolution, alternative imaging channels and the ability to customise light wavelengths are achieved (Bio-DOME). This is accompanied by an interactive calibration procedure that is robust to changes in the hardware configuration and provides fluorescence imaging (Fluoro-DOME). These alterations to the fundamental design allow for long-term use of the DOME in an environment of higher temperature and humidity. Thus, long-term imaging of living cells in a wound, with closed-loop control of real-time frontier illumination via projected light patterns, is facilitated.

**Supplementary Information:**

The online version contains supplementary material available at 10.1007/s12213-024-00165-0.

Introduction To study complex biological systems, long-term imaging of live cells is essential to visualise the location of cells, the intracellular mechanisms and the dynamic collective behaviours between cells and their environment. While visualisation is crucial, it is also important to establish effective methods to interact with cells through external stimuli. In cell biology, this is often achieved using chemical induction, however, optical stimulation is also widely used. Examples include photodamage to eliminate tumour cells [1], or to upregulate genes, e.g. p53, causing downstream effects such as accelerated cell migration [2], and optogenetics, which introduces a light-responsive construct into the cell to control cellular activity [3, 4].

Optical stimulation is often achieved using laser light from an imaging system [5] meaning that only one small area can be targeted at one time. It is often manually controlled by an operator, limiting the number of experiments which can be done. Conversely, the Dynamic Optical MicroEnvironment (DOME), which we have built, allows for the parallel illumination of many microscale cells simultaneously through the use of a digital light projector (DLP). The DOME controls light in space and time, allowing for precise and dynamic light patterns [6]. The DOME has three distinct modules: 1) an imaging module taking the form of a Raspberry Pi camera, 2) a projection module in the form of a Texas Instruments DLP and 3) an on-board computation module provided by the Raspberry Pi 4 board which conducts image analysis, turns LEDs on and off and sends light patterns to the projector.

Wijewardhane et al., demonstrated, for the first time, that a modified DOME can be operated from within an incubator [7] that maintains the environment at 37^∘^C, 5% CO_2_, and >90% humidity [8], in order to image live mammalian cells. Improved calibration of the camera and projector enabled light patterns to be projected onto a detected wound edge in real-time (Bio-DOME). Here, we present further modifications to the DOME that allow fluorescence imaging, improved calibration between the camera and projector that is user-friendly, enabling more precise illumination, and the ability to project the light patterns on the cells in a closed-loop fashion (called the Fluoro-DOME). This enabled autonomous and dynamic illumination of the leading edge of a healing wound, where the light pattern changes in line with what the camera observes. This example is based on the work done by Kozyrska et al., where they demonstrate that manually directed light-induced DNA damage at the leading edge of a wound can upregulate the gene p53, which subsequently accelerates cell migration. Irradiation occurred once and the scratched edge was manually identified and selected to be irradiated by the authors, with the laser irradiating each cell individually in sequence. The DOME system could automate this process and irradiate all the necessary cells at the same time.

By closing the loop between cellular imaging and control, we can create automated solutions for the imaging and control of cellular populations in applications including wound healing [2], cancer therapy [9], antibacterial treatment [10], and to study biological systems using optogenetics where illumination can alter membrane permeabilisation, cell polarity, induce cell death, and activate intracellular signalling pathways [11, 12].

**Fig. 1:**
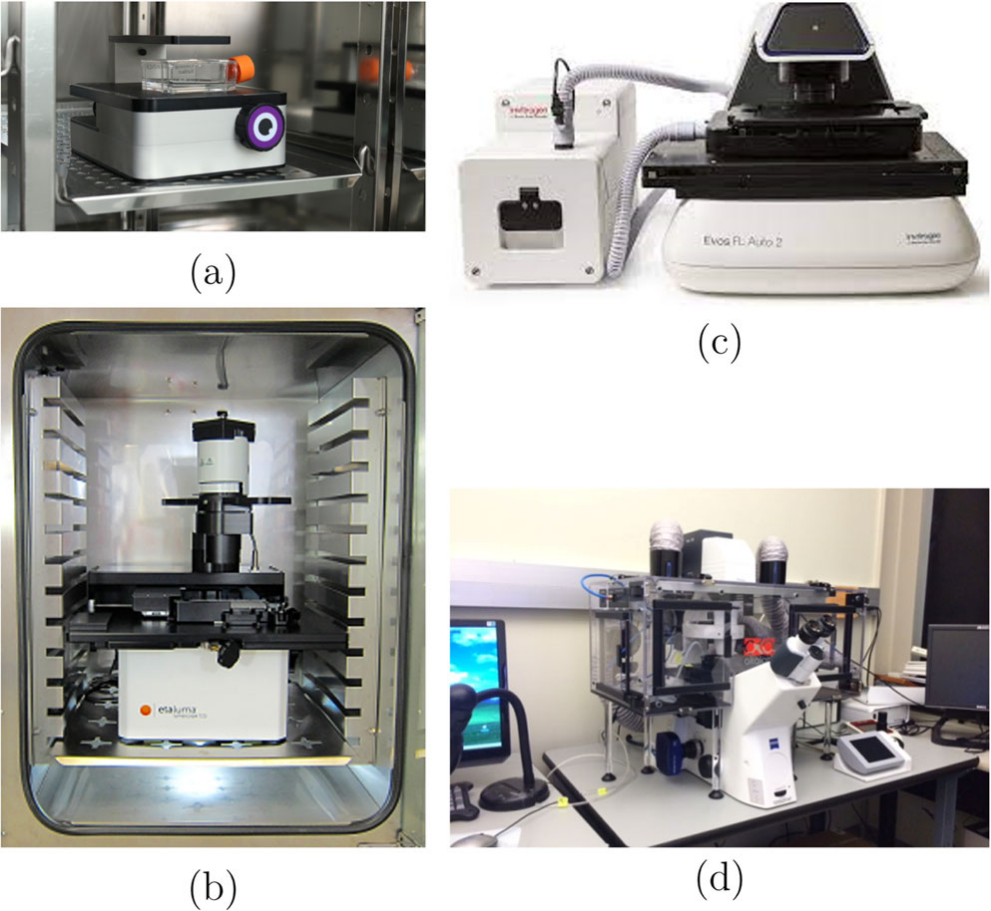
Examples of live-cell imaging microscopes. **(a)** Iolight’s compact inverted microscope sitting inside an incubator [13], **(b)** Etaluma’s inverted LS microscope [14], **(c)** EVOS Onstage Incubator system with an EVOS microscope [15], and **(d)** Okolab’s cage incubator around a microscope [16]

For comparison, commercial devices for live-cell imaging are grouped into three categories: incubator microscopes ($\pounds$1K-10K), microscopes with stage top incubators ($\pounds$10K-100K) and microscopes with cage incubators ($>$ $\pounds$100K). Incubator microscopes sit directly inside the incubator (Fig. 1a and b), for example, Iolight’s compact inverted microscope only has a single field of view [13], whilst Etaluma has an inverted microscope comparable to a traditional microscope [14]. Stage top incubators only create a chamber around the sample (Fig. 1c), such as Thermo Fishers EVOS Onstage Incubator (AMC1000) with EVOS M5000 and M7000 imaging systems [15], which are capable of imaging multi-wells and in fluorescence. Cage incubators create an environmental chamber around the microscope (Fig. 1d) and these systems have high specifications and functionality: multi-well imaging, different channels of fluorescence, objective choices, automated stages, and auto-focus. Okolab provides these cage incubators, which are adaptable to a range of microscopes [16]. But the high cost does make it inaccessible for some cell biology labs to have their own. Additionally, there are a variety of light illumination devices which have used holographic projection [17], spatial light modulation [18], and a structured illumination microscope [19], to conduct real-time light projection, however, they are also expensive and do not provide closed-loop control that causes the light to restructure in line with the sample changes.

For laboratories that conduct cell culturing and therefore have incubators, the Bio and Fluoro-DOMEs will function as an incubator microscope. From the commercial devices listed above, none of them are adaptable after purchase and are expensive if you require more than one function. Furthermore, none of these devices have the ability to control illumination patterns projected on the cells in a closed-loop manner. The DOME setup is modular allowing us to change the hardware, the wavelength used for projector illumination, and the imaging channel. All parts are low-cost, readily available, and the device is open source, allowing any lab to reproduce the setup for $\pounds$650 (Bio-DOME) or with fluorescence imaging added for less than $\pounds$1,000 (Fluoro-DOME) and continue adapting it to their specific needs.

**Fig. 2:**
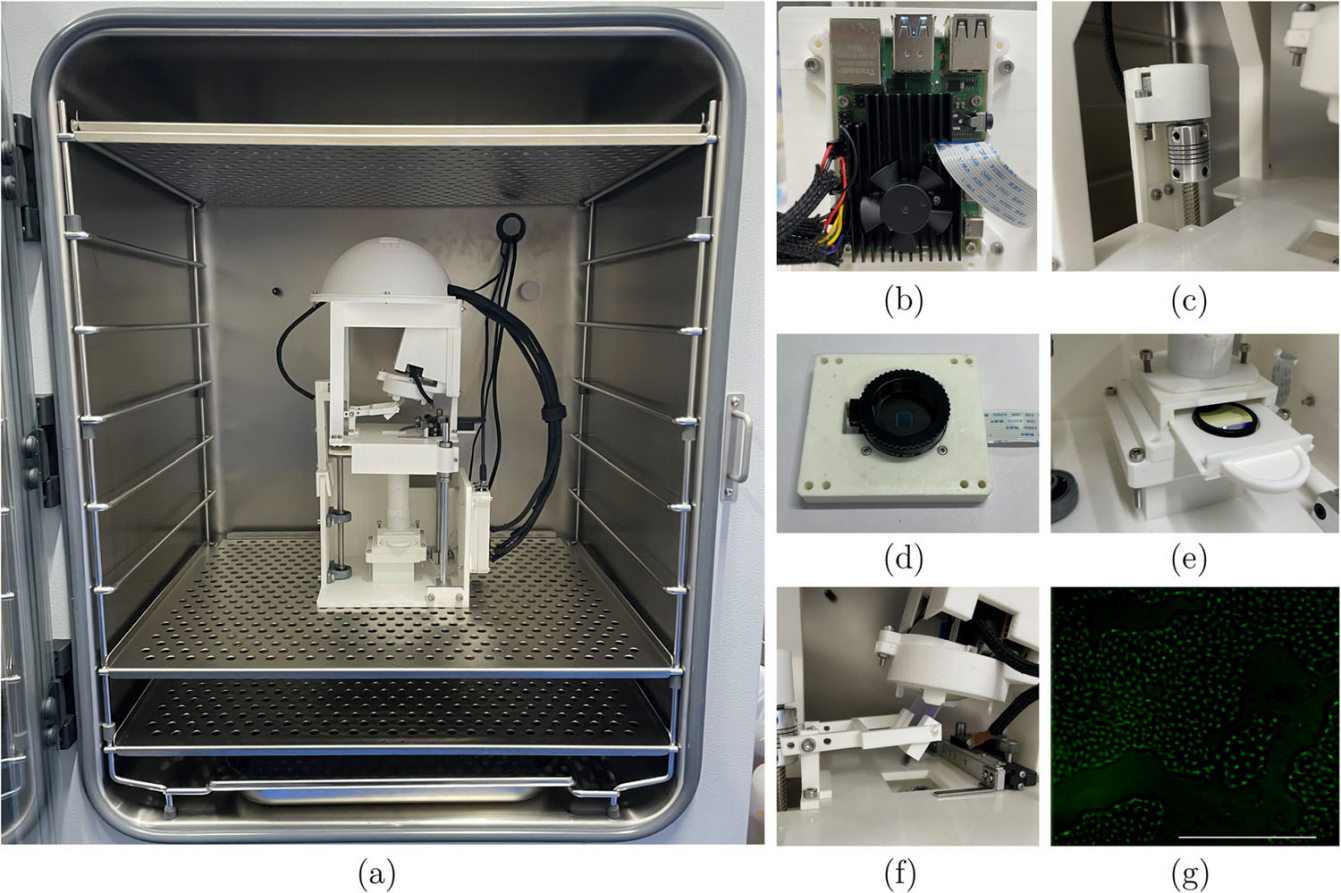
A breakdown of the modified and additional parts of the DOME. **(a)** The DOME inside an incubator. **(b)** A cooling fan attached to the Pi4, **(c)** close-up of the stepping motor to lead screw set up, **(d)** a high-quality camera with glass lens in the camera holder. **(e)** Close-up of the bandpass filter in the filter holder, **(f)** the fluorescence LED and dichroic mirror set-up, and **(g)** a fluorescence image of GFP-tagged MDCK cells. The scale bar is 0.5mm

## Methods

### DOME assembly

The DOME presented in our previous work [6] was adapted to work in hot and humid environments inside an incubator [7]. It is constructed by assembling 3D printed components in Acrylonitrile Butadiene Styrene (ABS) (Extrafill 2.85mm, Fillamentum), (Fig. 2a). The STL files are available on https://bitbucket.org/hauertlab/dome. The Raspberry Pi 4 (Model B 4GB, The Pi Hut) has an extreme cooling fan kit for Raspberry Pi (ZP-0042, 52Pi) was attached to the Pi4 via thermal tape onto the two central chips with a 5V and ground wire into the Pi4 GPIO pins (Fig. 2b).

A linear rod set (3D Printer Guide Rail Set, Glavanc) and linear motion ball bearings (LM8LUU) join the base and the sample stage together and via the lead screw, the Z-plane can be moved either upwards or downwards to enable sample focus. A stepper motor (28BYJ-48 5V DC) was attached to the lead screw and to the Pi4 board via a motor driver (ULN2003), allowing the Z-plane to be controllable from outside the incubator environment (Fig. 2c). The X-Y plane is controlled by a microscope calliper (Microscope Moveable Stage, Zetiling) attached to the sample stage. A breakdown of the cost of the DOME presented here can be found in the Appendix, totalling $\pounds$650 or with fluorescence imaging attached for $\pounds$997.

**Fig. 3:**
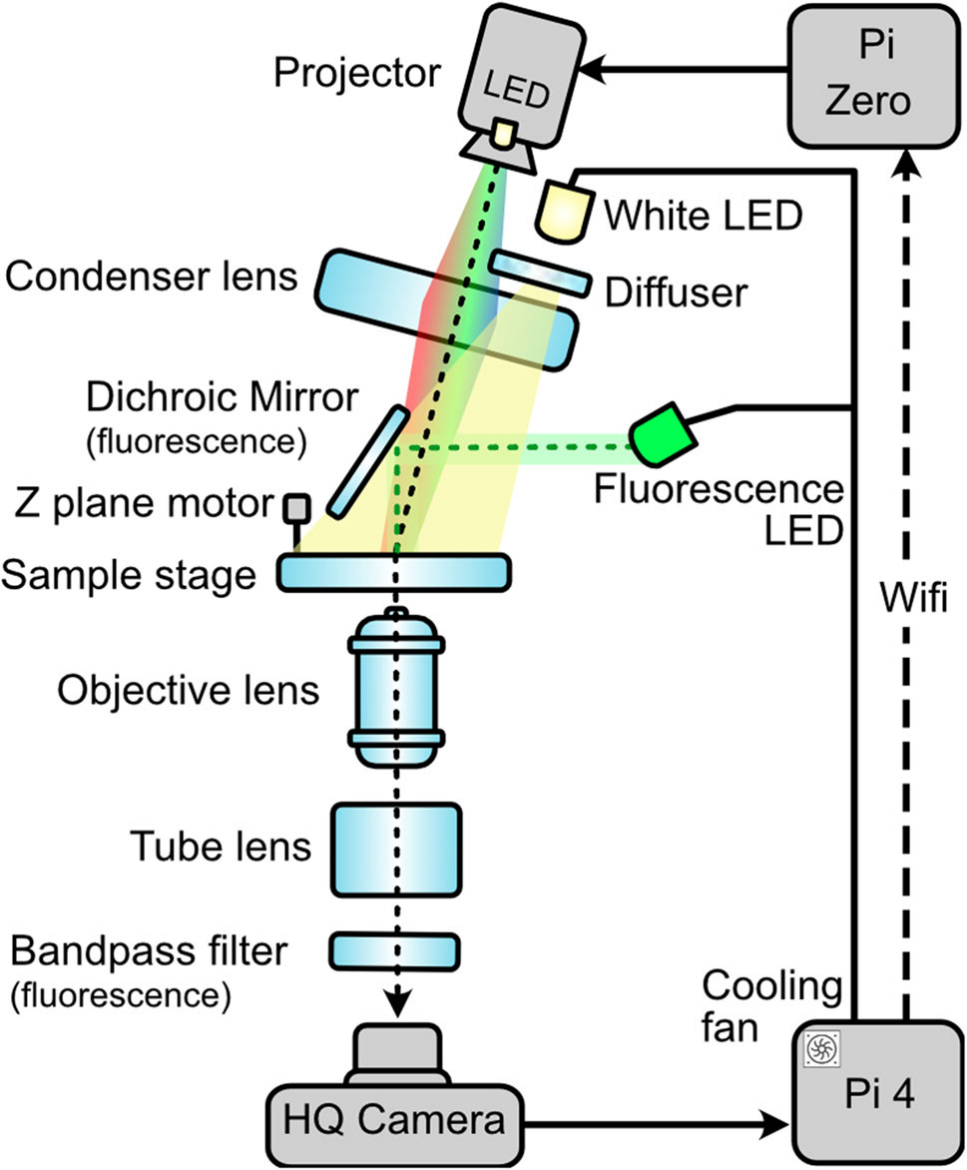
Schematic of the main components of the DOME. New components include HQ camera, cooling fan, Z-plane motor, changed light source in the projector and fluorescence imaging. The black dotted line indicates the light path from the projector and the green dotted line indicates the light path from the fluorescence LED. The dashed line represents the WiFi communication between the Pi4 and Pi Zero, and the bold lines show physically linked components. Figure adapted from [6]

**Fig. 4:**
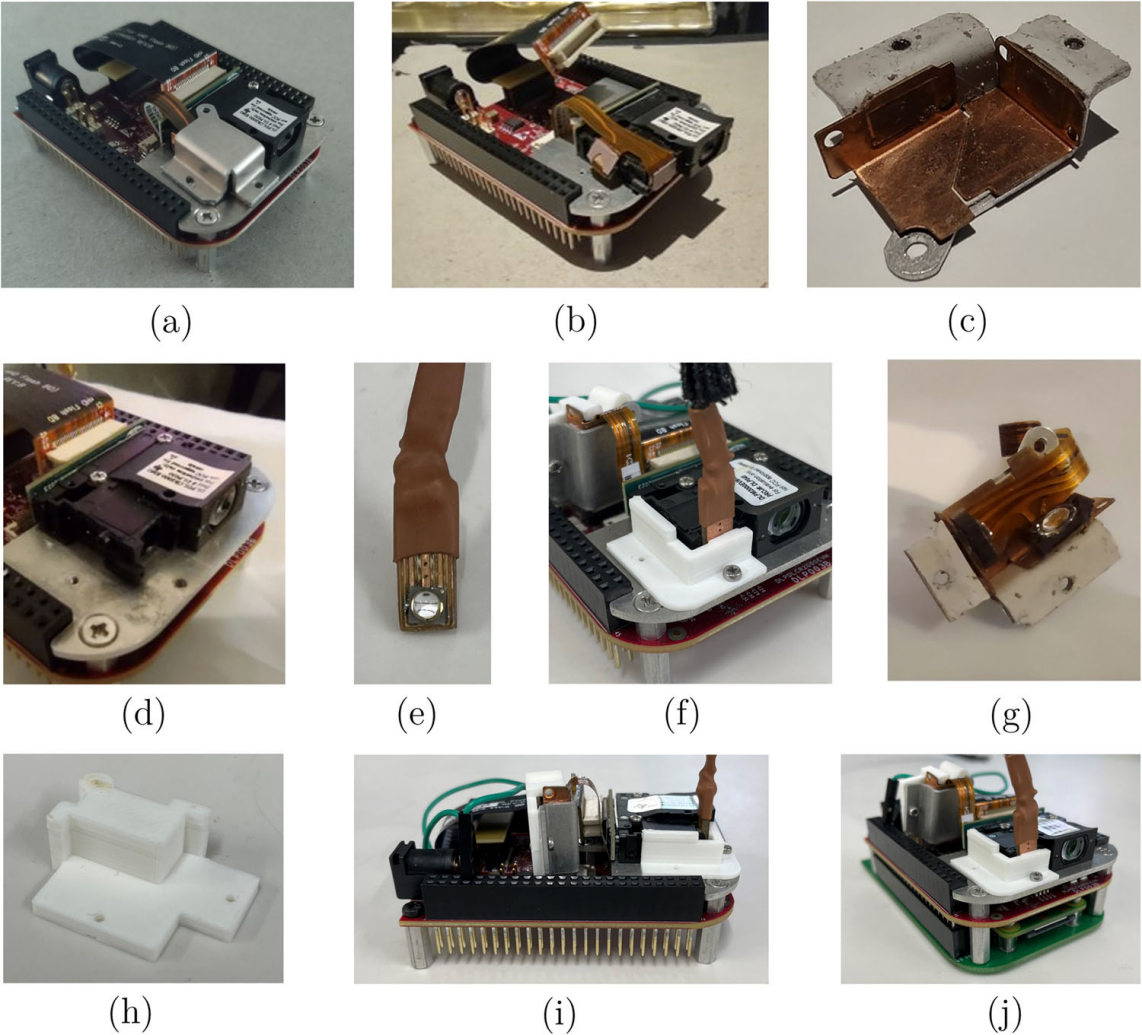
Changing the light source in the DLP. **(a)** Stock DLP projector board, **(b)** DLP projector with heat sink removed, **(c)** heat sink, **(d)** DLP lens housing with heat sink and RGB LEDs removed, **(e)** near-UV LED mounted onto a custom PCB, **(f)** projector with UV LED installed, **(g)** RGB LEDs with heat sink, **(h)** mount for RGB LEDs, **(i)** complete modified DLP projector, **(j)** DLP projector attached to custom interface PCB with Pi Zero

### Optical imaging setup

To image the cells in brightfield, a white LED (3.7 V 5 mm Through Hole, RS Components) is controlled by the Pi4 board through a condenser lens (50mm Diameter PCX, Edmund Optics) and a ground glass diffuser (DG10-1500, Thor Labs) to illuminate the sample stage (Fig. 3). The camera (Raspberry Pi High Quality Camera Module, The Pi Hut) sensor is protected by a circular glass (25 mm diameter uncoated gorilla glass window, Edmund Optics) and rubber seal (O-Ring, 23.5 mm Bore, 25.5 mm Outer Diameter, RS Components) (Fig. 2d). The camera images through an imaging column containing an objective lens (10X Standard Objective, Edmund Optics) and a tube lens (9X Eyepiece Cell Assembly, Edmund Optics). The camera is controlled by the Pi4 board and is connected by a camera connector (Flex Cable for Raspberry Pi Camera 300 mm, The Pi Hut).

For green fluorescence imaging, a blue 485nm LED (XPEBBL-L1-0000-00301, RS Components) mounted on a custom PCB is controlled by the Pi4 and the light is reflected off a 490 nm dichroic mirror (DMLP490R, Thor Labs) to excite the cells on the sample stage (Fig. 2e). A 520nm bandpass filter (FBH520-10, Thor Labs), which is the emission filter, was placed in between the camera and the tube lens (FIg. 2f), to image the emission of the green fluorescence protein (GFP)-expressing cells (Fig. 2g). An excitation filter was not required as the LED is at the specific wavelength needed to excite GFP.

### Replacing projector light source

**Fig. 5:**
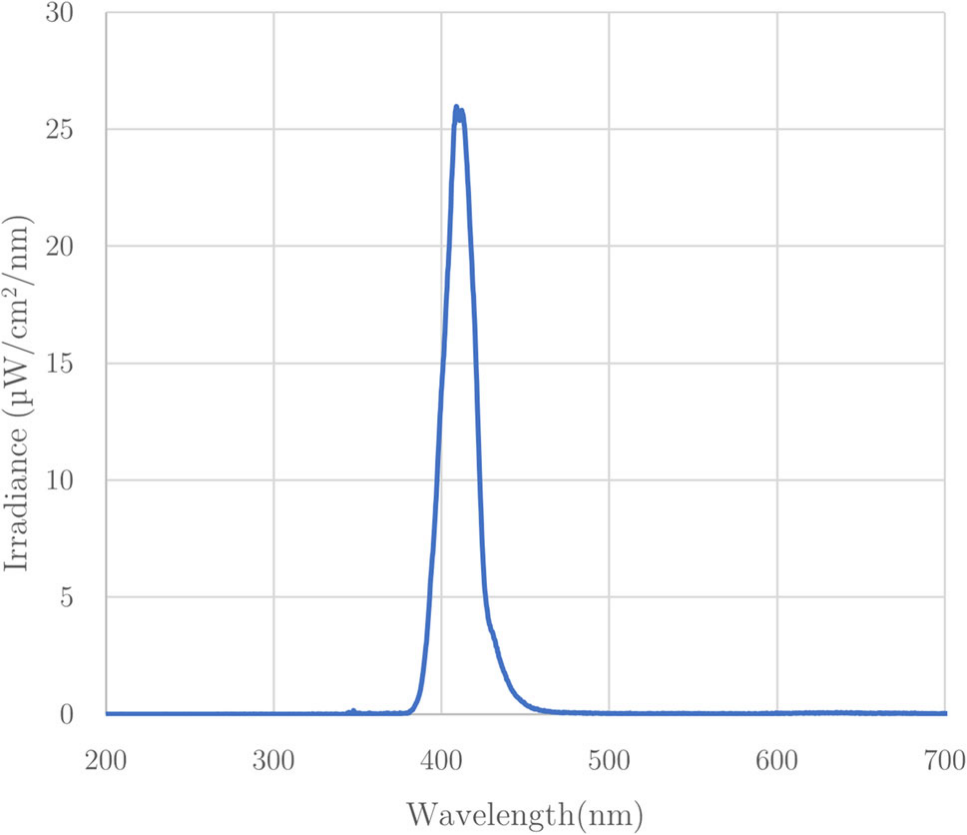
Spectrophotometry measurements are taken from the projector with near-UV LED. The relative radiance per wavelength of the LED through the projector and condenser lens

Different cellular systems react to different wavelengths. Here, we show how the light source of the projected light patterns can be modified to a LED with the desired wavelength. In the DOME, the projecting module contains a digital light projector (DLP Lightcrafter Display 2000 Evaluation Module, Texas Instruments), a Raspberry Pi Zero W (The Pi Hut) and a custom interface PCB (Pi Zero W adaptor board, Tindie). The original light source of the native DLP is a set of RGB LEDs, which we replaced with a near-UV 405 nm LED (VLMU3500-405-060, Vishay); Fig. 4 shows the DLP at various stages of modification. To access the lens slot for incoming light, we remove the heat sink from the projector board and detach the flexible PCB strip that contains the native RGB LEDs. Next, we 3D print a custom bracket to hold the new near-UV LED in place where the original blue LED would be. Finally, it was found that the DLP projector board contains a feature that prevents regular operation when the native LEDs are not electronically connected to the board. To overcome this issue, we designed a simple mount for the RGB LEDs that allows them to be safely concealed with the stock heat sink from the projector. This allows the RGB LEDs to remain electronically connected to the projector, thus allowing light patterns to be projected, whilst no longer functioning as the actual light source. The near-UV LED that replaces them can be operated using the standard GPIO pins on the Pi4. Light spectra measurements were taken with calibrated OCEAN-HDX (Ocean Optics/Insight). The measurement fibre with a cosine corrector was placed at the sample stage. Figure 5 confirms the peak wavelength of the near-UV LED is unchanged when measured through the projector and condenser lenses, as well as the corresponding radiance of the LED through the whole system.

### Redesigning calibration algorithm

To perform targeted illumination and closed-loop control, the mapping from pixel coordinates in the camera frame to the respective pixel coordinates in the projector frame that correspond to (viewing or illuminating) the same physical locations in the sample space must be known. Here, calibration refers to the process used to calculate this mapping in the form of a geometric transformation.

**Fig. 6:**
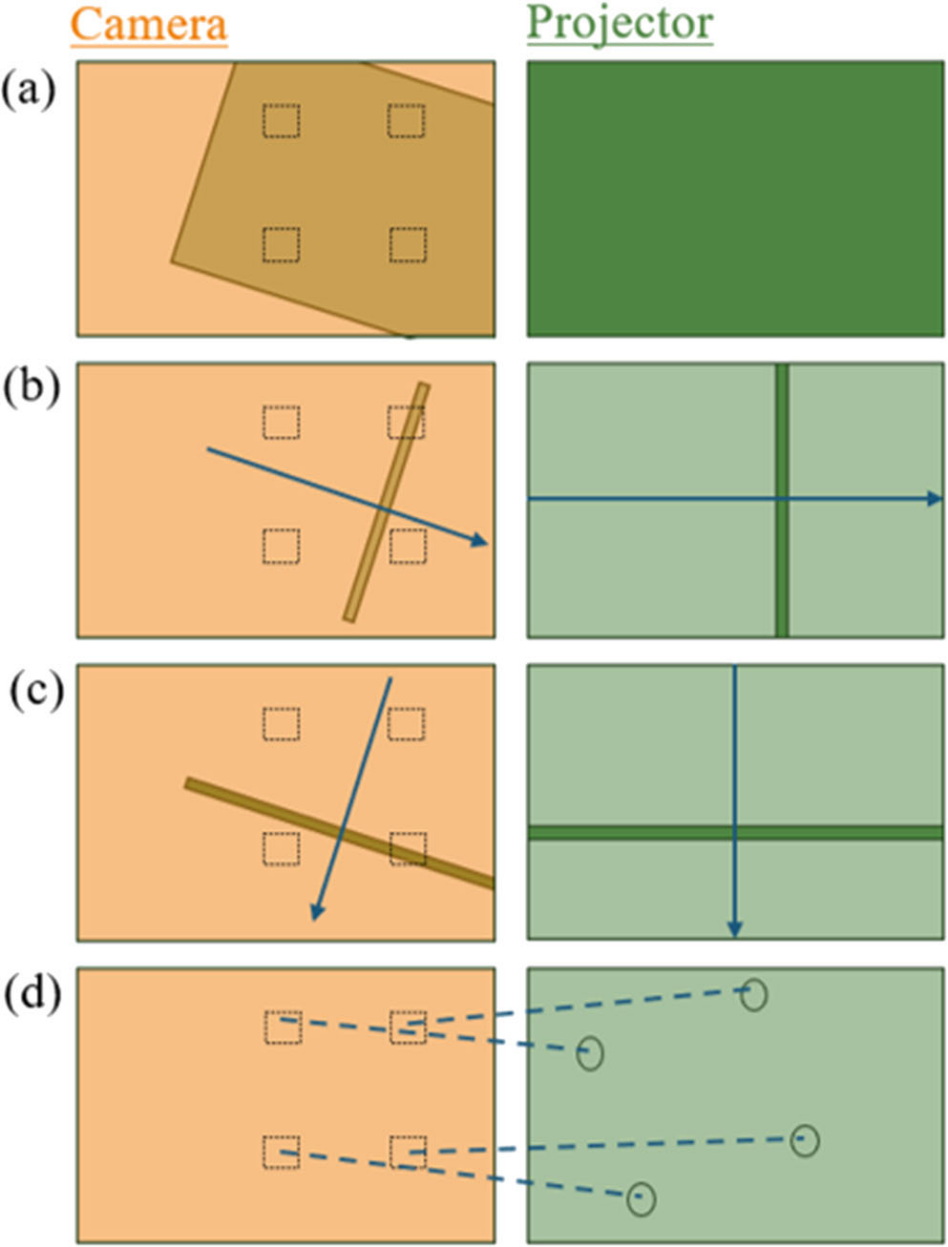
Illustration of main steps in the new calibration procedure, showing the perspectives of each reference frame. This example shows a generalised scenario where the projector is only able to illuminate a subsection of the sample space that can be viewed by the camera, and the camera is only able to view a subsection of the sample space that the projector can illuminate. Dotted squares show the locations of scan zones and circles show their estimated positions in the projector frame; corresponding pairs are linked with dashed lines

The original calibration algorithm for the DOME did not account for the non-zero angle between the normal vector of the sample space and the vector of the projector’s line of sight (see the angle between the dotted lines from projector to sample and from sample to the camera in Fig. 3) [6]. Furthermore, it is assumed that the relative angle between the reference frames about the Z-axis is no greater than 90^∘^. Whilst this does not present any practical issues with the standard DOME configuration, it does reduce the generality of the transformation that is found. A new calibration algorithm was proposed by Wijewardhane et al., in the Bio-DOME to address these limitations [7]. This consisted of the following steps:

1. Illuminate all pixels in the projector frame to detect the illuminable region of the camera frame, then evenly distribute scan zones in this region, Fig. 6a.
2. Sequentially illuminate sets of adjacent pixel columns in the projector frame, recording the intensity measured by the camera in each of the scan zones, Fig. 6b.
3. Repeat step 2 for rows of pixels in the projector frame instead of columns, again recording the measured intensities in each of the scan zones, Fig. 6c.
4. Estimate the positions of the scan zones in the projector frame by identifying the columns and rows that gave the highest measured intensities, Fig. 6d.

Once the corresponding pairs of scan zone locations are known, the transformation can be calculated in the form of an affine matrix using the computer vision Python library (*cv2* was used by Wijewardhane et al., [7]).

**Fig. 7:**
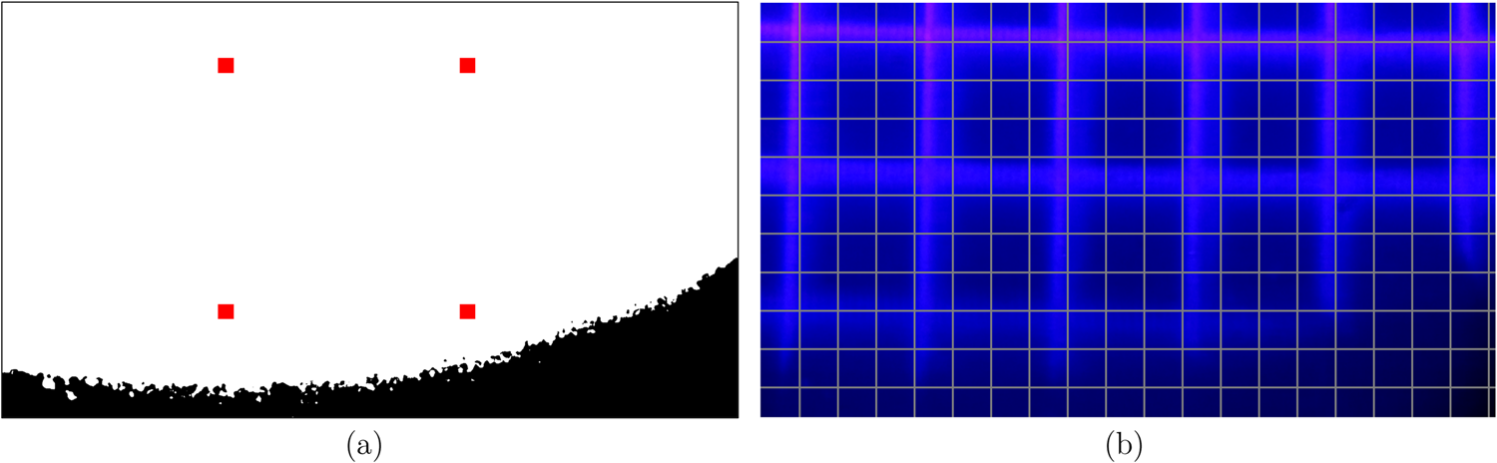
Example of visual feedback presented to the DOME user during calibration (see Fig. 6a). **(a)** Illuminable region that has to be detected with the user’s specified parameters (white pixels). The black pixels show regions of the camera frame that cannot be illuminated effectively by the projector. The red markers show the proposed centre positions of scan zones. The scale of the red markers is not representative of the actual size of the scan zones used during calibration; this is determined in subsequent steps. **(b)** Camera frame image of projected grid pattern (blue/purple) in the illuminable region shown in (a). A second reference grid with known spacing is superimposed on the image (grey). Using the relative sizes of the grid spacing, the level of magnification between the projector frame and the camera frame can be estimated

Whilst this procedure generalises to arbitrary camera-projector configurations well, it is not clear how certain parameters should be adjusted based on the exact hardware setup being used. For example, the strength of the magnification lenses could affect the optimal size of the scan zones. We present some additional augmentations to adapt hyper-parameters used in the calibration process. This is implemented as an interactive, text-based user interface. Firstly, during step 1 (see above), the user is asked to specify the brightness of the projector frame and the threshold to be used to detect the illuminable area in the camera frame. This allows the user of the DOME to find parameter values that are appropriate for the light source and camera module being used. An example of the visual feedback shown to the user can be seen in Fig. 7a. Note that the illuminable region that is detected in this image is not necessarily optimal, and is shown purely for the purpose of demonstration. In reality, with the correct hardware configuration, our current DOME setup can illuminate the entire camera frame.

In order to minimise the influence of small errors, the position of scan zone centres should be placed as far apart as possible. However, placing scan zones close to the border of the illuminable region could increase the likelihood of detecting false positives from light artefacts during steps 2 and 3 (see above). To compromise between these effects, a border variable $0 \le b \le 1$ can be specified by the user to control the relative distance between scan zone centres and the border of the illuminable region. When $b = 0$, scan zones are placed directly on the border, and when $b = 1$, scan zones are placed adjacent to each other in the centre of the illuminable region. Here, $b = \frac{1}{3}$ was found to be an effective value for our setup.

Before proceeding to steps 2 and 3 (see above), we estimate the optimal breadth of the camera frame scan zones, $s_c$. Ideally, this should allow at least 2 observed scan lines to intersect with the scan zones; if only a single intersection occurs, the calibration cannot be accurately determined. Thus,

1 $$\begin{aligned} s_c \approx 2 l_c \quad , \end{aligned}$$

where $l_c$ denotes the breadth of the scan line as observed from the camera frame. This is related to the breadth of the scan line in projector frame pixels $l_p$, according to,

2 $$\begin{aligned} l_c = M l_p \quad , \end{aligned}$$

Here, we ask the user to specify the value for $l_p$, whilst advising that lower values will generally improve accuracy but increase computation time during the scanning steps. $l_p = 10$ was found to be sufficient for our setup. In order to estimate the level of magnification before the calibration mapping has been calculated, the relative sizes of squares in two grids can be compared, as depicted in Fig. 7b.

The user can manually change the pixel spacing used for the projected grid $G_p$, until several grid squares are clearly visible. By allowing the user to input the observed size of a projected grid square, $g_p$, relative to the size of a square in the super-impose camera frame grid, $g_c$, the magnification can be approximated by solving,

3 $$\begin{aligned} \frac{g_c}{G_c} \approx M \frac{g_p}{G_p} \quad , \end{aligned}$$

for *M*, where $G_c$ is the known pixel spacing of the super-imposed camera frame grid. Finally, solving Eqs. 1, 2 and 3 leads to,

4 $$\begin{aligned} s_c \approx \frac{ 2 l_p g_c G_p}{G_c g_p} \end{aligned}$$

By setting the breadth of the scan zones according to Eq. 4, the calibration can proceed with hyper-parameters that are optimised for the DOME user’s setup.

**Table 1:** A summary of the different versions of the DOME system

	DOME	Bio-DOME	Fluoro-DOME
Paper	[6]	[7]	Micro and Bio Robotics
Camera	V2 (8 MP)	HQ (12 MP)	HQ (12 MP)
Illuminable Wavelength (nm)	460, 510, 617	405	405
Light Intensity (mW)	280, x*, 450	6.8	750
Brightfield	Yes, via Projector	Yes, via White LED	Yes, via White LED
Fluorescence	No	No	Yes
Imaging Magnification	X9, X90 (X10**)	X10	X10
Z-Plane Control	Manual	Electronic	Electronic
Environment	RT 20^∘^C	37^∘^C Incubator	37^∘^C Incubator
Calibration Algorithm	Quadrant Illumination	Column Illumination	Column Illumination v2
3D Print Material	PLA	ABS	ABS
Cooling System	No	Yes	Yes
Camera Field of View (mm)	6x6, 1.875x1.875	2.38x1.38	2.38x1.38
Camera pixel at 10X ($\mu$m)	12x12, 3.75x3.75	1.25x1.25	1.25x1.25
Price ($\pounds$)	691	650	997

*Value not given in the datasheet

**In the biological field, the tube lens is not considered in the magnification, therefore X90 here refers to X10 and X9 is only the tube lens

## Results

### Summary of the DOME modifications

The Fluoro-DOME was built on previous versions of the DOME, including the initial DOME which provided the basis of spatio-temporal closed-loop control of micro-agents using light. Volvox, an algae was used as the micro agent in the original DOME. To be able to image and control living mammalian cells, the DOME has to be robust to high temperatures and humidity. The Bio-DOME sits inside a cell culture incubator, set to 37^∘^C, 5% CO_2_, and 90% humidity to allow for long-term imaging of cells. The Bio-DOME is constructed in ABS, which has a high glass transition temperature $T _{g}$ of $\sim$106^∘^C [20], suitable for the higher temperatures inside the incubator. Higher resolution imaging is possible by the Raspberry Pi High-Quality (12 megapixels) camera; the camera is humidity-proofed by placing a clear glass and rubber seal to protect the camera sensor. A stepping motor attached to the lead screw allows the z-plane of the sample stage to be controlled outside of the incubator, reducing the time the incubator door is open, and reducing the temperature, CO_2_ and humidity fluctuations inside the incubator. A cooling fan was attached to the Pi4 to keep the board at 40^∘^C and prevent overheating. During initial trials the Pi4 board was heating up to the upper limit when any script was run, this made long-term imaging unattainable. The cooling fan operates quietly and does not create a large airflow to avoid disturbing the cells or causing vibrations through the body of the Bio-DOME that would affect imaging.

To control living cells with light, we required wavelengths and intensities not provided by the native LEDs of the DLP. We therefore developed a method to fit custom LEDs and PCBs inside the incubator. Additionally, the calibration algorithm was slow, we improved the algorithm by using a column illumination method and took into account the angle difference between the projector and camera. The Fluoro-DOME provides a new channel of imaging in fluorescence; cells that are tagged with GFP, are excited at 488 nm light and then imaged at 520 nm (Fig. 2g). GFP is localised to the nucleus of the cell. Higher intensity LED in the projector and improved calibration algorithm enable light to be illuminated at better precision. Table 1 summaries the differences between the various published DOMEs.

### Ability to image living cells with the DOME

**Fig. 8:**
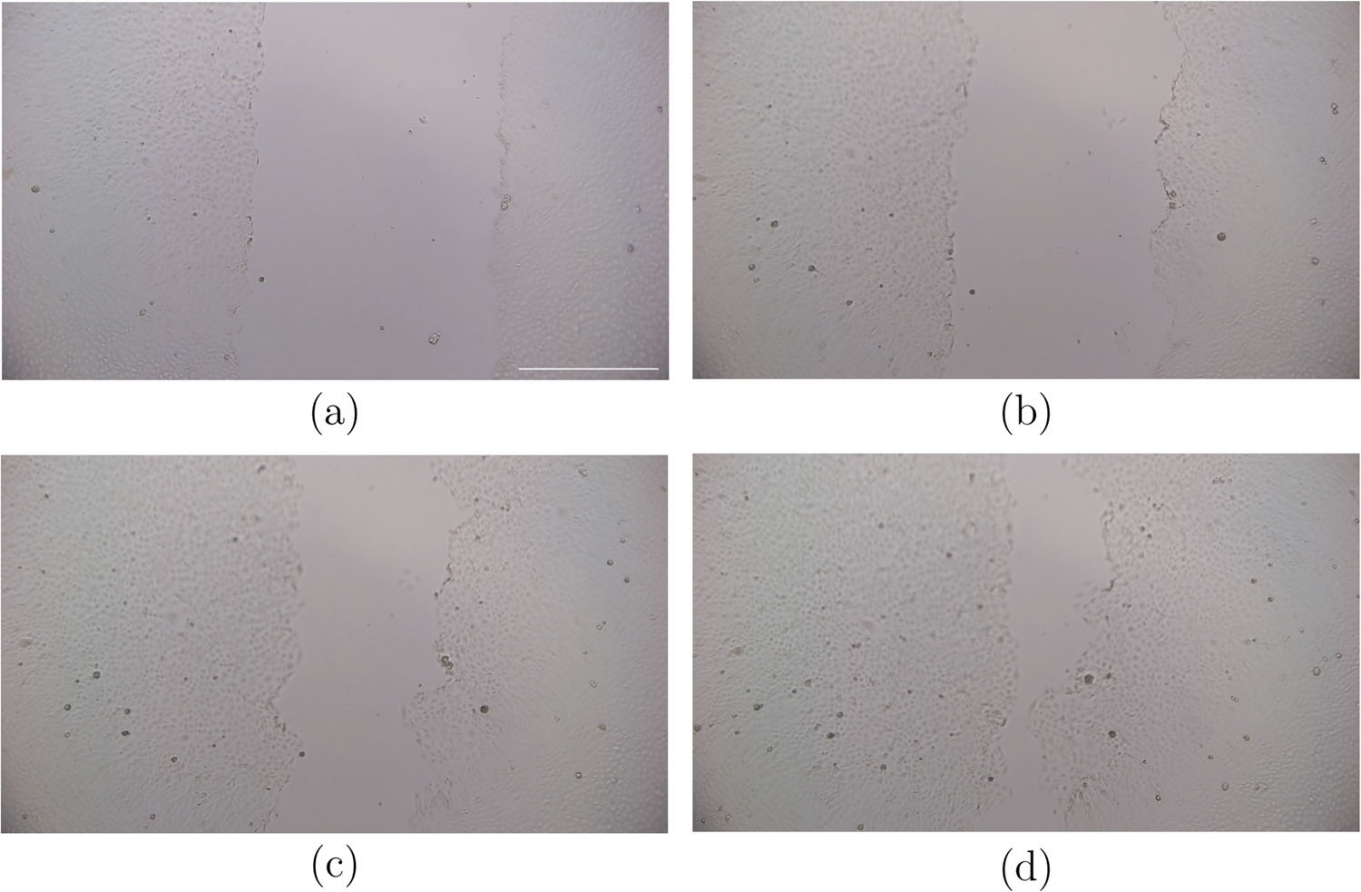
Scratch assay on Mardin Darby Canine Kidney (MDCK) cells. **(a)** 0 hour, **(b)** 5 hours, **(c)** 10 hours, and **(d)** 20 hours. The scale bar is 0.5mm. The full 24-hour time-lapse is in the supplementary

The DOME is now able to image time-lapses of living cells over prolonged periods inside an incubator and maintain the viability of cells (Fig. 8). Trials were run with images taken every 5 minutes for 24 hours. There was some focal drift throughout the time-lapse (see supplementary video 1), however, the cells are still visible to a good resolution. The images captured have a field of view of 2.38 x 1.38 mm when imaged at 10x magnification at a resolution of 1920 x 1088 pixels with no artefacts. This means an individual camera pixel represents a real-world area of 1.25 x 1.25 $\upmu$m. The DOME is also able to offer fluorescence imaging, here we have shown green fluorescence at 488nm wavelength. This enables distinct borders of the fluorescently tagged cells can be identified, which will assist with accurate image analysis and thus projector pattern creation and illumination (Fig. 2g).

**Fig. 9:**
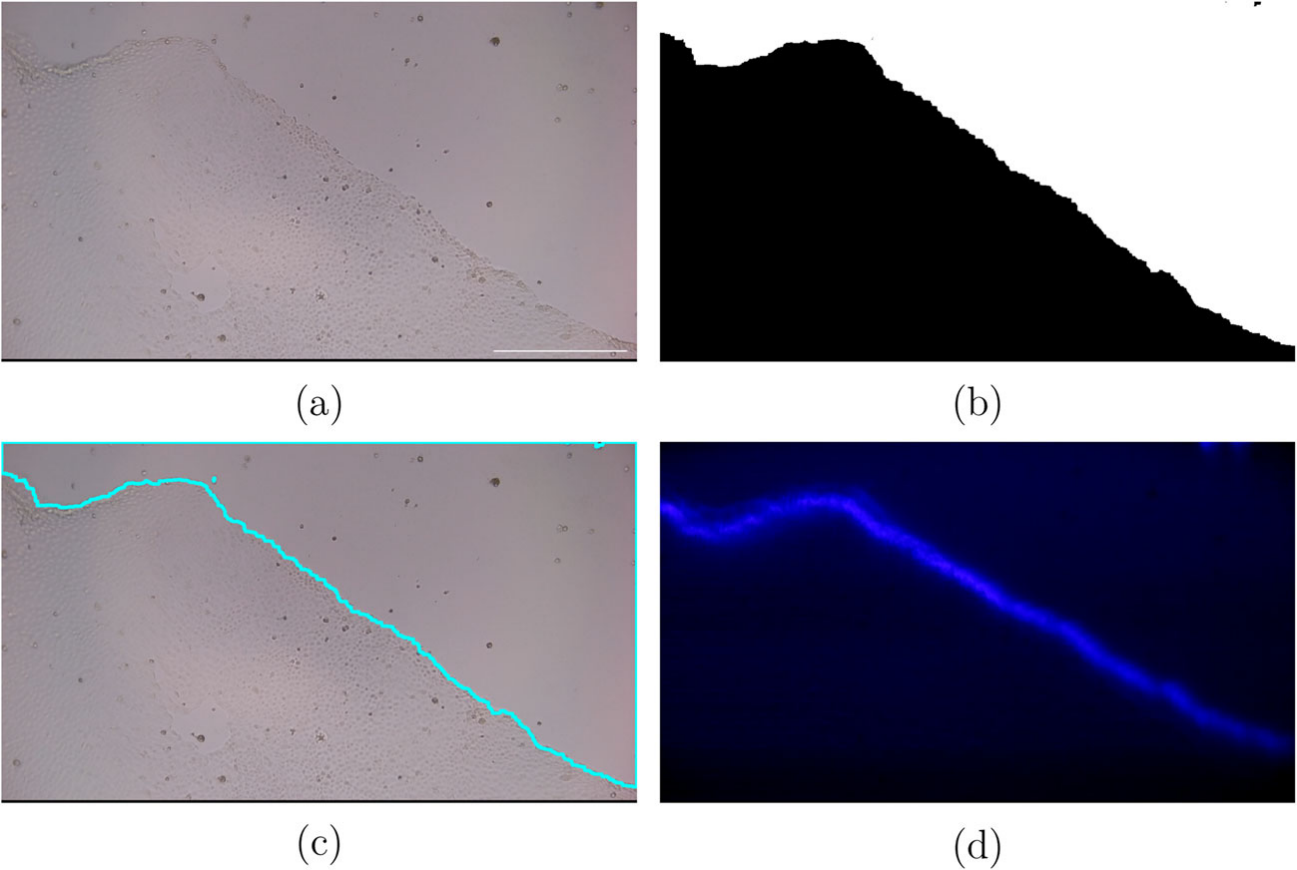
Wound edge targeting by light. **(a)** Brightfield image of MDCK cells with a wound. **(b)** image segmentation conducted by the Pi4, **(c)** contour created from the image segmentation, and **(d)** projector light pattern of the contour onto the MDCK cells. The scale bar is 0.5mm

### Targeting populations of living cells with light

As the DOME is able to image live cells at a high enough resolution and the camera is calibrated with the projector to image near-UV light, the DOME is now capable of illuminating cells in a targeted manner. We show a proof of concept that the DOME can be used to target the leading edge of a wound, similar to Kozyrska et al., but in an autonomous and closed-loop manner. In Fig. 9, we show a brightfield image taken of a scratched monolayer of Mardin Darby Canine Kidney (MDCK) cells (Fig. 9a); image analysis was conducted on the Pi4 which first contrasts the image (Fig. 9b), then identifies the longest continuous line as the leading wound edge, shown in light blue (Fig. 9c). The light blue line is converted into coordinates and these are sent to the projector, which makes the pattern to be projected (Fig. 9d). We take the illumination further by allowing the edge detection algorithm to be run repeatedly. This causes the projected light pattern to change in line with the changes in the sample, enabling the closed-loop control of the cells. This can be seen in the video in Supplementary S2, where light was projected every 30 minutes for 6 hours.

## Discussion

The DOME offers the ability to illuminate a sample with projected light patterns based on observations from real-time image analysis. Owing to the adaptations presented here, the DOME can now image live cells long-term inside an incubator. This is achieved using brightfield and fluorescence imaging with a high-quality camera and Z-plane control. The closed-loop localised illumination of cells using light is a novel addition to a microscope that increases the scope for spatio-temporal manipulation of mammalian cells. Further optimisations to the dosage and intensity of the 405nm light would enable light-induced acceleration of cell migration by causing localised DNA damage [2]. The irradiation area of the wound edge can be optimised, Kozyrska et al., discuss leader cells being the main cells at the leading edge that are responsible for advancing the migrating wound. Therefore, targeting just the leader cells with irradiation may provide a therapeutic alternative to irradiating the entire wound edge. This could open up new possibilities for automated therapeutic treatments that use targeted light doses to accelerate wound healing.

Although the DOME does not have the high specifications of many current microscopes used by cell biologists, it provides a unique environment for the spatio-temporal control of cells, where convenient live-cell imaging can also occur at a fraction of the cost. With now the addition of another imaging channel in fluorescence; we are able to identify individual cells and enhance the segmentation of cells. A limitation of this device is that an incubator is required, but if a lab is conducting cell biology they will require an incubator to store the cells and thus the DOME is compact enough to fit inside an incubator with room to house the cell stock.

The improved imaging resolution of the DOME achieved in this work takes the real-world area represented by a single pixel from 3.75 x 3.75 $\upmu$m to 1.25 x 1.25 $\upmu$m [6]. This 3-fold improvement greatly increases the range of possible agents that could be easily visualised and controlled by the system, including many bacteria [21], and at the smaller scale, microparticles [22] and micromotors [23]. Therefore the DOME has potential applications in fields outside of mammalian cell biology. The use of projected light to diminish biofilms in microbiology [24], optogenetic control in synthetic biology [25], or the use of photothermal effects in lipid chemistry [26]. As well as soft (polymer) micro-robotics where light can be used to cause mechanical manipulation to integrate polymers and biological organisms [27]. Hydrogels can be constructed to mimic the extracellular matrix enabling more accurate cellular microenvironments [28]. UV lithography allows for 3D micropatterning of hydrogels which can be used for rapid prototyping of various micro-structures [29, 30]. Usually, a photomask is used to develop the pattern, but the DOME could illuminate the specific pattern, eliminating the need for a photomask and possibly creating different layers of micropatterning in the hydrogel.

The next step in making the DOME a more robust device for live-cell imaging would be to provide phase contrast imaging, enabling the enhanced localisation of cells and sub-cellular structures, as well as making cell counting and tracking algorithms more accurate. As the DOME is able to image in fluorescence, we will need to mechanise the insertion of the bandpass filter between the camera and the tube lens. Currently, the filter is manually slotted in and needs to be removed to conduct brightfield and projector illumination. The camera is unable to see the 405 nm projection with the 520 nm bandpass filter, therefore mechanisation would allow quick changes between imaging channels, as well as being able to integrate fluorescence imaging into the closed-loop feedback. In this DOME, imaging with a 10x objective was the most appropriate magnification as we can visualise a larger field of view, but for other applications, higher magnification will be useful to visualise smaller agents. With the improved calibration procedure, the user can easily adapt hyper-parameters to changes in the hardware setup, such as using different levels of magnification. This allows the accuracy of the calculated mapping to be maintained when modular changes are made to the DOME’s hardware configuration.

The Z-plane could have a more sensitive motor so that the degree per step is smaller, allowing finer control. However, we did not want to use an external power supply, instead using the Pi4 board itself as the power source for the motor, and in this case, the Z-plane control is satisfactory. During long-term imaging, there was some focus drift, this could be mitigated by adding an auto-focusing camera, alternatively, we have programmed the stepping motor to hold the Z-plane in the desired position. The sample stage could be motorised to allow for programmable X-Y control, shortening the time the incubator door is open and allowing full control of the DOME from outside. The solution for replacing the projector light source could be further refined by bypassing the need for the RGB LEDs to remain electronically connected. This could involve a custom circuit that mimics the electrical resistances of the RGB LEDs, allowing for a modular projector light source without the RGB LEDs attached.

## Conclusion

The DOME is a unique platform that allows for closed-loop, localised light control of microscale agents while also providing visualisation in multiple channels. Here, this functionality was extended to include live-cell imaging through adaptations to the Fluoro-DOME’s modular design, making it suited to an incubator environment and capable of imaging at a higher resolution. A protocol was developed for switching out the native DLP LEDs to allow the choice of custom wavelengths for wavelength-specific applications. Taken together, these adaptations allow for the long-term imaging and light-based control of agents that require precise environmental conditions. This capability greatly increases the potential utility of the DOME system, particularly in the biological and medical realm.

### Supplementary Information

Below is the link to the electronic supplementary material.

Supplementary file 1 (avi 34202 KB)

Supplementary file 2 (avi 3011 KB)

## Data Availability

Data availability: Not applicable. Code availability: Code and STL files can be found in the Bitbucket linked in the Methods and Materials section.

## Appendix A

**Table 2:** Cost of the DOME for live-cell imaging and targeted light illumination, given to the nearest GBP at time of purchase ($\pounds$) and inclusive of 20% VAT)

Type	Cost ($\pounds$)
Optical	
Projector (Digital Light Projector)	109
Condenser Lens (50 mm Diameter PCX)	36
Tube Lens (9X Eyepiece Cell Assembly)	63
Objective Lens (10X Standard Eyepiece)	121
Camera Lens (25 mm Uncoated Gorilla Glass Window)	28
Camera Seal (O-Ring, 23.5 mm Bore, 25.5 mm Outer Diameter)	1
Glass Diffuser (DG10-1500)	15
Longpass Dichroic Mirror for GFP imaging (490 nm)	205
Bandpass Filter for GFP imaging (520 nm)	141
Electrical	
Raspberry Pi4 Board (4 GB)	54
Raspberry Pi Zero Board (Zero W)	11
Camera (Raspberry Pi High Quality Camera Module)	50
Camera Connector (Flex Cable 300 mm)	2
SD Card X2 (SanDisk Ultra 16 GB + 32 GB microSDHC)	13
Interface PCB (Pi Zero W Adapter Board)	3
Power Supply (Raspberry Pi4 Power Supply)	8
Power Supply (Plug-in Power Supply 5V)	12
Cooling Fan (Extreme Cooling Fan Kit for Raspberry Pi)	14
Stepping Motor (28BYJ-48 5 V DC)	5
Motor Driver (ULN2003)	2
Brightfield LED (3.7 V White)	1
405 nm LED (VLMU3500-405-060)	6
485 nm LED (XPEBBL-L1-0000-00301)	1
Mechanical	
ABS Filament (Extrafill 2.85 mm Filament)	19
Linear Rail Set (Glvanc 3D Pinter Guide Rails)	28
X-Y Calliper (Zetiling Microscope Moveable Stage)	22
Linear Motion Ball Bearing (LM8LUU)	7
Fastening and Wiring (Assortment)	20
Total	997
